# *Mycobacterium avium* Lymphadenopathy among Children, Sweden

**DOI:** 10.3201/eid1404.060570

**Published:** 2008-04

**Authors:** Johanna Thegerström, Victoria Romanus, Vanda Friman, Lars Brudin, Paul D. Haemig, Björn Olsen

**Affiliations:** * Kalmar County Hospital, Kalmar, Sweden; † Linköping Medical University, Linköping, Sweden; ‡ Swedish Institute for Infectious Disease Control, Stockholm, Sweden; § Sahlgrenska University Hospital, Göteborg, Sweden; ¶ Kalmar University, Kalmar, Sweden; # Uppsala University, Uppsala, Sweden

**Keywords:** Nontuberculous mycobacteria, Mycobacterium avium, child, epidemiology, season, water, dispatch

## Abstract

We studied *Mycobacterium avium* lymphadenopathy in 183 Swedish children (<7 years of age) from 1998 through 2003. Seasonal variation in the frequency of the disease, with a peak in October and a low point in April, suggests cyclic environmental factors. We also found a higher incidence of the disease in children who live close to water.

*Mycobacterium avium* is the most common of the nontuberculous mycobacteria that infect otherwise healthy children (*1*). It manifests as a chronic granulomatous lymphadenopathy in the neck region that preferably is treated by excision of the affected lymph node (*2*). In Sweden the incidence of nontuberculous mycobacterial disease in children increased from 0.06/100,000 population to 5.7/100,000 after discontinuation of the general BCG (Bacillus Calmette-Guérin) vaccination program in 1975 (*3*).

*M. avium* is common in the environment. It has been isolated from natural and human-made water systems as well as from soil and animals (*4*,*5*). The role of animals in its epidemiology is not clear. The main hypothesis is that oral contact with *M. avium–*infected water causes lymphadenitis in the head and neck region (*4*). An environmental study in nearby Finland found nontuberculous mycobacteria in 100% of brook water samples (*6*).

Human populations who live close to water have a higher proportion of persons with positive reactions to *M. avium* and other nontuberculous mycobacterial sensitins (*7*,*8*). In Sweden, 25.4% of non-BCG–vaccinated schoolchildren in an urban coastal area reacted to *M. avium* sensitin (*7*). In studies from Canada and the United States/Canada, *M. avium* disease in children has previously shown no seasonal variation (*9*), or it has been dominant in winter, spring, or both (*1*,*2*).

In Sweden, nontuberculous mycobacterial infection has been a reportable disease since 1989; all cases are reported to the Swedish Institute for Infectious Disease Control. From 1998 through 2003, 186 culture-positive cases of *M. avium* infection in children <7 years of age were reported. We registered age and residence and reviewed the children’s medical records to establish the month of onset of disease (date parent discovered an enlarged lymph node). Permission to conduct the study was obtained from 6 of the 7 ethical committees in Sweden. We collected information from 127 patient records. To these we added 35 cases that occurred during 1983–1997 (*3*,*10*). One patient with lung infection and 2 children whose home addresses could not be located were excluded. Altogether, 183 children (1998–2003) were included in the geographic analysis, and 162 children (1983–2003) with culture-proven *M. avium* lymphadenopathy were included in the analysis of seasonal variation.

Sweden is divided into 290 urban and rural districts. The exact number of children <1–6 years of age in every district is known for each year during 1998–2003. Each district was evaluated by using a map with a scale of 1:300,000. Within each district we estimated the proportion of the total child population 1) living within 5 km of the coast or a big lake or within 2 km of a small lake or river and 2) living in the different cultivation zones 1–8 (zone 1 being the warmest, as defined by the Swedish association for leisure gardeners). A cultivation zone and a water category (salt or brackish water, fresh water, or no water) were assigned to each case-patient according to the home addresses of the children. We assumed most children were infected in the area where they resided.

Incidence rates were calculated and the corresponding 95% confidence intervals estimated by using the Poisson distribution, since events that occur randomly in time follow this distribution. Results of seasonal variations were tested with a commercial statistical software (Statistica version 7.1; Statsoft, Tulsa, OK, USA), by using nonlinear regression fitting a sine function (Figure 1).We considered p values <0.05 statistically significant. (See also online Appendix Table, available from www.cdc.gov/EID/content/14/4/661-appT.htm)

**Figure 1:**
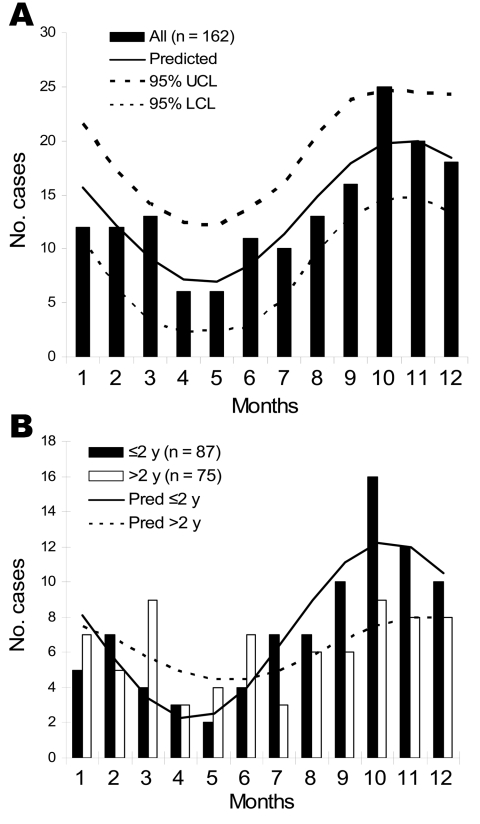
Seasonal incidence of *Mycobacterium avium* infection in Swedish children (1983–2003) in our study (bars = real numbers) and as predicted by nonlinear regression sine functions (equations: y = a + bsin[(x – c)Π/6], where x represents the months (1–12) (www.smhi.se), and with “a,” “b,” and “c” characteristic for each curve and b ≠ 0 with statistical significance, p<0.05, for all these curves. (See also online Appendix Table, available from www.cdc.gov/EID/content/14/4/661-appT.htm) A) All children. The curves were statistically significant, p<0.05, for both 1983–1997 and 1998–2003, and so the data for all years were grouped together. UCL, upper confidence limit; LCL, lower confidence limit. B) Children <2 years and >2 years of age, respectively. “b,” amplitude of curve, has a tendency to be greater for children <2 years of age (p = 0.07) and “c” is slightly smaller for children >2 years of age, representing a shift to the right of the curve, though not statistically significant. Pred, predicted.

The overall annual incidence rate of culture-verified *M. avium* lymphadenopathy in children <1–6 years of age in Sweden during 1998–2003 was 4.5/100,000 children. Seasonal variation was statistically significant (p<0.05), with a peak in October and a low point in April (Figure 1). The mean time from onset of symptoms at home to diagnostic puncture at a hospital was 4.3 weeks. The incubation period is unknown. From in vitro growth data, we assumed that the time of infection was 2–8 weeks or longer before clinical manifestations. Seasonal variation tended to be more accentuated in children <24 months old (Figure 1). Our interpretation is that younger children might have shorter incubation periods (weeks) because of their immature immune systems (*11*), whereas the incubation periods of older children might be longer (weeks to months) and more variable, resulting in a shift to the right and a flattened sinusoidal curve. Thus, the curve of the data for the younger children would be closer to an imagined curve of “true” inoculation time.

We found no significant difference in seasonal variation when we compared colder (*4*–*7*) and warmer (*1*–*3*) cultivation zones. Because of compensation after the vernal equinox of more light in the North, the beginning of meteorologic spring and summer differs no more than 1 month between cultivation zones (www.smhi.se). Spring means better temperature and nutritional conditions for growth of bacteria in nature. *M. avium* can enter a metabolic state of dormancy in response to starvation and recover rapidly when conditions improve again (*12*). We speculate that the observed seasonal variation is due to a combination of changing temperature and nutritional conditions in the environment throughout the year as well as changing human activities in different seasons. The reason why previous studies have not shown this seasonal variation might be due to a smaller number of investigated cases (*9*), the inclusion of more heterogeneous materials (older children and other mycobacteria beside *M. avium*), or conduction of studies in regions with less clear-cut seasonal variations than Sweden.

Our results also show a correlation between *M. avium* clinical disease in children and living close to water (Figure 2). Socioeconomic status usually is associated with living close to water. In Sweden, however, socioeconomic differences in society are small and therefore not likely to be a confounding factor. Correlation to water has previously only been incidental—indirectly implied by studies of sensitin reactivity in healthy populations (*7*,*8*) and by the isolation of *M. avium* from natural and man-made water systems (*4*). However, 1 Swedish sensitin study, conducted in an urban area situated by the sea, disagrees with our results. It found a high sensitivity to *M. avium* PPD (*7*), whereas the incidence of clinical disease in our material is low in the same area. We speculate that the reason for this could be that inhalation of *M. avium*–containing aerosols might be sufficient to convey sensitin positivity but that acquisition of the clinical disease demands close contact with and oral ingestion of natural water, which are less likely to happen to a child who lives in a city. Seasonal variation and higher incidence in regions near natural fresh water compared to inland areas and cities indicate that the source of *M. avium* transmission is outdoor natural water rather than tap water.

**Figure 2:**
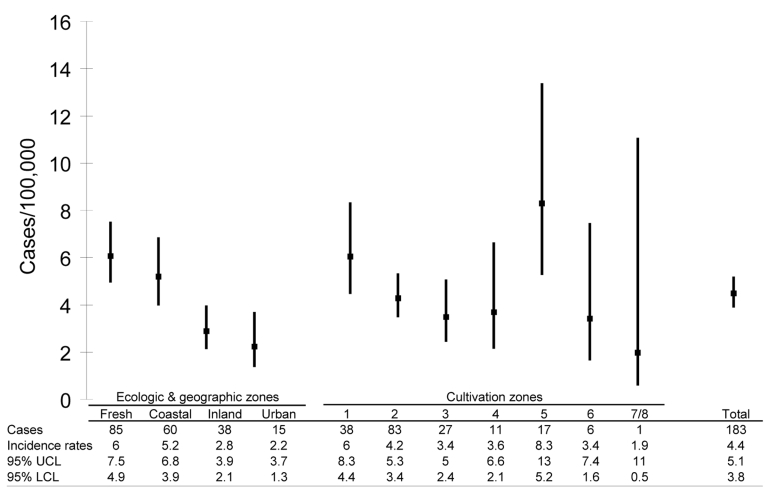
Number of cases, incidence rates (cases/100,000 children/year), and 95% confidence intervals of *Mycobacterium avium* disease in children grouped according to ecologic, geographic, and cultivation zones, Sweden, 1998–2003. Freshwater, coastal (incidence of saltwater and brackish water were similar within this group), inland, urban (Stockholm, Göteborg, and Malmö, the 3 largest cities in Sweden) areas and the different cultivation zones (1–8, zone 1 being the warmest) are depicted. When assigning zones to each case, we assumed that the children were infected in the area where they resided. UCL, upper confidence limit; LCL, lower confidence limit.

We found an extremely high incidence of *M. avium* lymphadenopathy in a few contiguous districts of cultivation zone 5 in northern Sweden, where the human population has historically been isolated and where some genetic diseases exist (*13*). The higher frequency of *M. avium* lymphadenopathy in this exceptional area raises the possibility that development of clinical disease requires a genetically predisposed host. Children with localized *M. avium* lymphadenopathy might not have the known and more severe impairment of γ-interferon–mediated immunity (*14*), but their immune status should be further investigated for milder defects.
